# The Typical Computed Tomography Findings of Primary Fallopian Tube Carcinoma

**DOI:** 10.2174/0115734056274106240119052437

**Published:** 2025-06-18

**Authors:** Tongtong Tian, Rongrong Ding, Tongmin Xue, Jun Sun, Jun Ling

**Affiliations:** 1 Department of Radiology, Northern Jiangsu People’s Hospital, Medical School of Yangzhou University, Yangzhou 225001, Jiangsu Province, China; 2 Center of Reproductive Medicine, Northern Jiangsu People’s Hospital, Medical School of Yangzhou University, Yangzhou 225001, Jiangsu Province, China

**Keywords:** Fallopian tube, Primary fallopian tube carcinoma, Computed tomography, MSCT, Pelvic pain

## Abstract

**Aim:**

This study aimed to investigate the imaging features of primary fallopian tube carcinoma (PFTC).

**Methods:**

Imaging findings of 12 PFTC patients were retrospectively studied. Multi-slice computed tomography (CT, MSCT) was performed to investigate tumor location, size, density, appearance (cystic/solid), enhancement pattern, and metastasis.

**Results:**

Twelve women aged 34–67 (mean=54.3) years were presented with pelvic pain (n=6), vaginal discharge (n=5), and incidental pelvic masses (n=3). The tumor diameters of PFTC varied from 3.3 to 6.8 cm (mean=4.7 cm). Ten cases were unilateral, and two were bilateral. The lesions were adnexal tubular-shaped cystic masses with mucosal papillary nodes in six cases, irregular cystic and solid masses in four cases, and sausage-shaped solid masses in two cases. The plain CT values ranged from 15 to 35 HU (mean, 28 HU). On enhanced CT, the enhancement of the solid composition was lower than that of the myometrium in all phases. CT values in arterial and venous phases were 55-62 and 60-63 HU, respectively, with average values of 58.6 and 61 HU. The metastasis sites included the ovary (n=2), omentum (n=3), retroperitoneal lymph nodes (n=5), pelvic lymph nodes (n=5), and inguinal lymph nodes (n=2). Seven cases exhibited pelvic fluid, and seven exhibited round ligament thickening on the lesioned side.

**Conclusion:**

In patients presenting with vaginal discharge or genital bleeding and sausage-shaped or tubal-shaped cystic, solid, or solid-cystic complexes in the adnexal portion associated with hydrosalpinx and peritumoral ascites, PFTC should be considered in the diagnosis, especially in tumors associated with round ligament thickening.

## 1 INTRODUCTION

Primary fallopian tube carcinoma (PFTC) is a rare tumor accounting for 0.3% of all gynecologic malignancies [1]. The incidence of fallopian tube carcinoma has increased in recent years [1-3], indicating that the incidence of PFTC might have been underestimated. Therefore, more attention should be paid to PFTC. However, radiological diagnosis of PFTC remains difficult. The difficulty in diagnosing PFTC early often results in poor prognosis. Thus, this study aimed to review the characteristic imaging findings of PFTC, thereby enabling radiologists not only to diagnose but also to aid clinicians in staging, treatment planning, and surveillance for recurrence.

## 2 MATERIAL AND METHODS

### 2.1 Patients

The institutional review board of our hospital (Northern Jiangsu People’s Hospital) approved this study, and informed consent was obtained from all the participants. We retrospectively reviewed the picture archiving and communication system (PACS) and pathological data and identified 12 PFTC patients between 2010 and 2020. The patient characteristics and clinical symptoms are presented in Table **1**. Lower abdominal pain, genital bleeding, watery discharge, adnexal mass, and other symptoms were not found in patients #5, #4, #2, 5 and #1 of the 12 patients, respectively.

### 2.2 Computed Tomography (CT) Acquisition Protocol

Examinations were performed using 64 detector row scanners (LightSpeed VCT 64; GE Healthcare, Milwaukee, WI, USA). Iodinated intravenous contrast material (iopromide, Ultravist 350; Schering, Berlin, Germany) was administered at a dose of 2.5 mL/kg of the weight and a rate of 2.8 mL/s. A plain scan was performed first, followed by arterial and venous phase scanning with 30 and 45 s delay, respectively, and fourth scanning with a two-minute delay phase.

### 2.3 Imaging Analyses

Two experienced imaging specialists (J SUN and J LING, with 25 years of experience in CT) were responsible for image analysis. The tumor position, size, shape, and margins were evaluated. The presence of solid or cystic portions, density, and enhancement was also recorded. The presence of lymph nodes, metastasis, and invasion was also documented. Consensus was reached after discussion if disagreement arose between them.

## 3 RESULTS

### 3.1 CT Findings

Twelve patients underwent preoperative imaging. The lesions ranged from 3.3 to 6.8 cm (mean diameter, 4.7 cm). Ten patients had unilateral disease, and two patients had bilateral disease. Six and eight tumors were located in the right and left adnexal regions, respectively. Twelve patients presented with irregular solid, cystic, or solid-cystic complex adnexal masses. Six patients presented with adnexal cystic structures with multiple solid mural nodules and papillary projections. The cystic structures appeared sausage-shaped (n=4), or C-shaped (n=2), or S-shaped (n=2). The solid mural nodules exhibited mild enhancement in the arterial and venous phases on contrast-enhanced CT. Four patients presented with mixed cystic and solid components that showed heterogeneous enhancement on contrast-enhanced CT scans. Two patients had an adnexal, sausage-shaped, predominantly solid mass separated from the ovaries. On contrast-enhanced CT, the solid component exhibited mild enhancement in the arterial and venous phases. Reinforcement of the solid component was less than that of the uterus.

Metastases were confirmed in ten cases by postoperative histopathological examination. The metastatic sites were the ovary in two, the omentum in three, retroperitoneal lymph nodes in five, pelvic lymph nodes in five, and inguinal lymph nodes in two cases. Pelvic fluid was associated with seven cases. Seven cases were associated with the thickening of the round ligament on the lesioned side. The round ligament extended naturally and showed homogeneous enhancement on the enhanced CT images. Pathological examination revealed adenocarcinoma, including well-differentiated adenocarcinoma (five patients), poorly differentiated adenocarcinoma (three patients), and moderately differentiated adenocarcinoma (four patients). Hematoxylin- and eosin-stained sections of each specimen were examined. Histologic evaluation was based on the classification of malignant epithelial fallopian tube carcinoma by the World Health Organization. Grading was performed according to the system established for ovarian epithelial carcinoma. The pathological diagnosis and histologic type of PFTC were confirmed independently by two experienced gynecologic pathologists. The levels of serum tumor markers (CA-125) in all patients were examined before surgery. The CA-125 levels in four cases were within the normal range. CA-125 levels increased significantly in five cases and were slightly elevated in three cases.

### 3.2 Patient 1: A 61-year-old Woman with PFTC

**Fig. (1):**
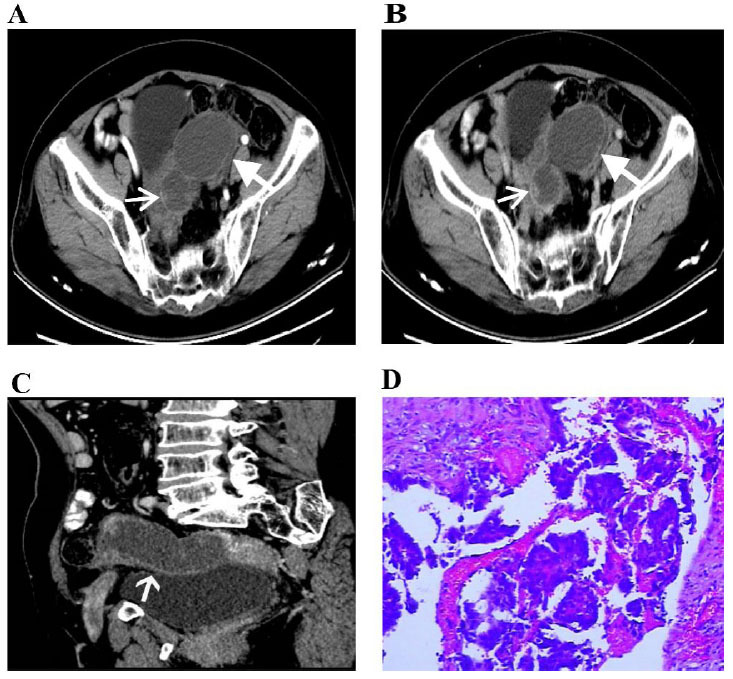
Primary fallopian tube carcinoma in a 61-year-old woman. Computed tomography images (**A**–**C**); HE staining shows histopathological patterns (HE×100) (**D**).

Axially enhanced CT showed a multilocular cystic structure in the left adnexal area. A small nodular soft tissue component (narrow arrow) was observed within the dilated fallopian tube. The degree of enhancement of the solid components was less than that of the uterus in the arterial phase. The multilocular cystic structures are demonstrated as a dilated fallopian tube (wide arrow) (Fig. **1A**-**B**). Oblique multiplanar reconstruction (MPR) CT revealed the left adnexal sausage-shaped cystic structure (white arrows) with mural nodule connected to the uterus (Fig. **1C**); photomicrography revealed papillary growth of tubal mucosa composed of columnar cells with high nucleus-to-cytoplasm ratio (HE×100) (Fig. **1D**).

### 3.3 Patient 2: A 54-year-old Woman with PFTC

**Fig. (2):**
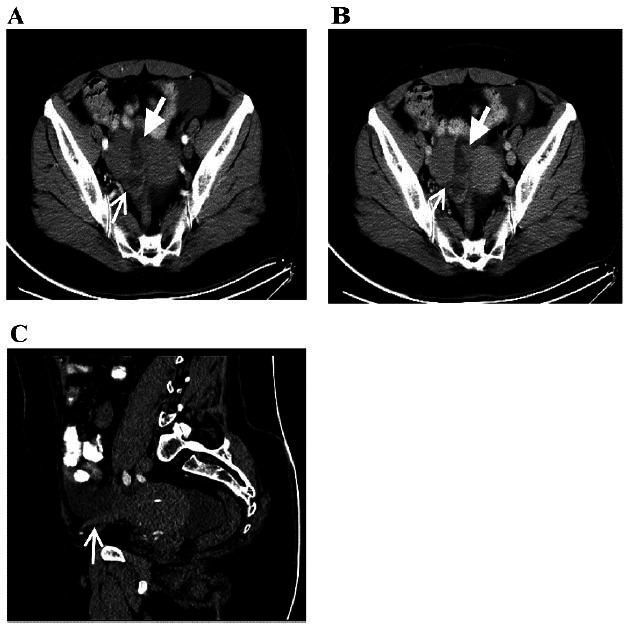
Primary fallopian tube carcinoma in a 54-year-old woman. Computed tomography images (**A**–**C**).

In the arterial phase, a solid cystic adnexal mass with small mural nodules demonstrated mild enhancement (narrow and wide arrows) (Fig. **2A**). The solid components and nodules exhibited lower enhancement than that exhibited by the uterus in the venous phase (Fig. **2B**). Non-enhanced CT sagittal MPR revealed the thickening of the ipsilateral round ligament. The white arrow indicates the thickening of the round ligaments (Fig. **2C**).

### 3.4 Patient 3: A 61-year-old Woman with PFTC

**Fig. (3):**
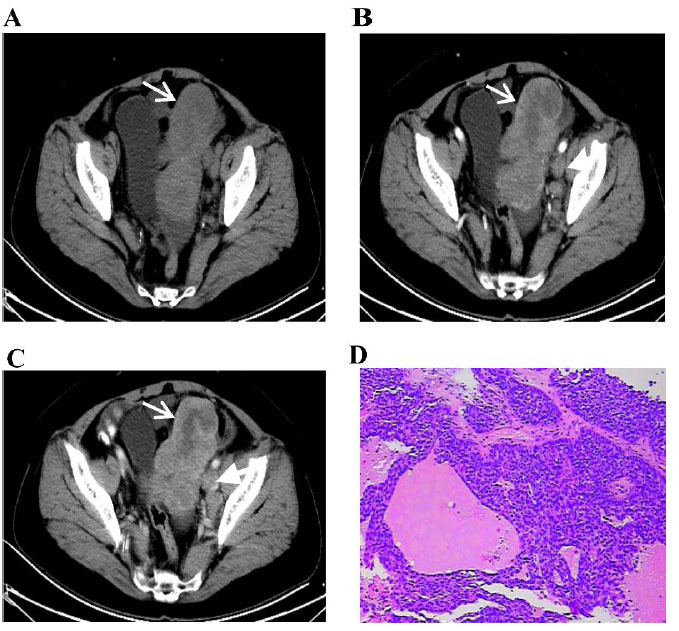
Primary fallopian tube carcinoma in a 61-year-old woman. Computed tomography images (**A**–**C**); HE staining reveals the histopathological patterns (HE×100) (**D**).

Axial non-enhanced CT demonstrated a sausage-shaped adnexal mass connected to the left uterine corner (Fig. **3A**). Enhanced CT demonstrated a solid component showing mild enhancement in the arterial phase (white arrow). Enlarged lymph nodes were found beside the iliac vessels (black arrow) (Fig. **3B**); the solid portion representing the true tumor exhibited lower soft tissue density enhancement than that of the myometrium. The cystic portion was serpentine, which might have indicated a hydrosalpinx (white arrow) (Fig. **3C**). Large infiltrating epithelial nests were observed in the tissues; the focal areas were glandular, and necrosis was common (HE×100) (Fig. **3D**).

## 4 DISCUSSION

Primary fallopian tube carcinoma is rare, accounting for approximately 0.3% of all female genital tract malignancies [2]. The predisposing factors for this malignancy are unknown; however, they are associated with infertility and pelvic inflammation [3, 4]. PFTC most commonly occurs in patients between 40 and 60 years of age, with a median age of 55 years (range 17–88 years). It is often insidious and nonspecific in presentation. Latzko’s triad of classic symptoms is vaginal bleeding or watery discharge and lower abdominal pain or an adnexal mass, but only 6–15% of PFTCs present with these features [5, 6]. In the current case series, only two patients (16.7%) presented with the classic triad of symptoms.

PFTC with papillary features is the most common histological type [6], with serous carcinomas being the most common histological subtype. The CA-125 antigen is often expressed and elevated; however, elevated serum CA-125 levels have been detected more frequently in epithelial ovarian carcinoma [7]. CA-125 levels represent a nonspecific diagnosis of fallopian tube cancer. CA-125 can be used to evaluate the treatment response and is a useful tumor marker for detecting tumor recurrence [8, 9]. Although PFTC has been well-described in pathological studies, imaging studies are scarce. Owing to the low incidence of PFTC, awareness of its existence is low, which hinders its imaging diagnosis. Although a precise diagnosis based on CT findings alone is difficult, we identified several unique features of PFTC on CT.

Imaging findings of PFTC have been described in a few reports. In these cases, PFTC presented as a cystic adnexal mass or a solid cystic mass that was difficult to distinguish from ovarian tumors. Reviewing the literature and the 12 cases in our study, the CT signs of PFTC were divided into three categories depending on the presence or absence of hydrosalpinx and the morphological characteristics of the solid tumors [9]. PFTC usually originates in the mucosal layers of the ampulla, and its growth pattern can be nodular, papillary, infiltrative, or mass-like. The tumor produces copious amounts of serous fluid, which accumulates and distends the tube, resulting in tubal dilatation [4]. The CT manifestation of PFTC depends on the presence or absence of a hydrosalpinx, and a solid tumor is the dominant component [9-11]. If only a small amount of fluid is produced or if the fluid depressurizes the uterus or enters the peritoneum [5], the solid tumor is the dominant component that is completely located within a dilated fallopian tube not associated with hydrosalpinx [10]. Solid tumors usually appear as sausage-shaped masses or intraluminal papillary nodules within the fallopian tubes. In our study, two patients showed a solitary solid mass confined within a dilated fallopian tube. One patient was presented with a small round mass surrounded by a small multilocular cystic portion in the ampulla, indicating a dilated tube. If the tumor is relatively confined to the fallopian tubes and tends to produce large amounts of serous fluid, the hydrosalpinx is considered the main component of it. The tumor usually presents as a fluid-filled tubular adnexal structure containing nodular or papillary solid components. Hydrosalpinx can present as a sausage-shaped, C-or S-shaped tubular cystic mass with multiple solid mural nodules and may also present as a multilocular cystic mass with a spoke-wheel appearance. This spoke wheel appearance can be attributed to the folding of the dilated tube, particularly when it is massively dilated. With such an appearance, PFTC is indistinguishable from ovarian epithelial tumors. Three patients in our study had sausage-shaped or C-shaped adnexal cystic masses with multiple solid mural nodules. The circuitous cystic mass is inseparable from the ovary and connected to the uterine horn on serial imaging. The sausage-like circuitous cystic mass was demonstrated to be a dilated fallopian tube after surgery [11, 12]. If the presence and ratio of the hydrosalpinx are equal to the solid components of the tumor, the whole lesion becomes a huge mass with mixed cystic and solid components interposed between the uterus and ovary.

These morphological characteristics of the solid-cystic complex on radiological examination represent the pathological features of fallopian tube tumors. Primary tubular tumors grow inside the lumen, appear circular or oval, and are usually sausage-shaped. The solid area was the same as that of the lesions discussed in the previous case, and the cystic area suggested duct dilatation [2]. In this study, four cases exhibited lateral fallopian tube dilation with irregular wall thickening and soft fusiform or oval masses. Ovarian cancer typically presents as a large cystic mass with varying amounts of solid components [12, 13]. Our series misinterpreted these three lesions as ovarian cystadenocarcinomas. On serial imaging, the cystic area might be interpreted as a loop of a dilated fallopian tube based on its characteristic serpentine shape [2, 14].

The attenuation on unenhanced CT depends on the presence of hydrosalpinx and the distribution or proportion of solid tumors. On a plain CT scan, PFTC appears as a mass with attenuation similar to other pelvic soft tissues. Plain CT values range from 15 to 35 HU, averaging 28 HU. According to the published literature, the solid portion of PFTC is characterized by low vascular impedance [9, 13]. On contrast-enhanced CT, the mural nodule or intraluminal mass exhibited slight arterial phase enhancement and less venous phase enhancement. The solid composition of the PFTC enhancement was lower than that of the myometrium during all phases [15].

The round ligament of the uterus comprises smooth muscle, connective tissue, blood vessels, nerves, and the lymphatic system [16]. Tumors arising from the round ligament are relatively rare. Moreover, to our knowledge, this is the first study to report on PFTC associated with the thickening of the round ligament. In the current study, seven cases exhibited thickening of the round ligament on the affected side, which exhibited histologically normal fibrous connective tissue. The cause of round ligament thickening cannot be clearly explained. However, we believe that the continued and prolonged compression of the ligament may obstruct the lymphatic system and result in the thickening of the ligament [11, 16]. The round ligament of the lesion extended naturally and presented homogeneous enhancement on contrast-enhanced CT images. In our experience, thickening of the round ligament is a particularly important sign of PFTC, which can help identify and differentiate it from other pelvic masses.

## CONCLUSION

If a patient presents with vaginal discharge or genital bleeding and a sausage-shaped or tubal-shaped cystic, solid, or solid-cystic complex is revealed in the adnexal portion and is associated with hydrosalpinx and peritumoral ascites, PFTC should be included in the differential diagnoses; especially, when the tumor is associated with thickening of the round ligament. This study, however, was limited by its retrospective design and the small number of patients, which limited the generalizability of our findings. Therefore, further research is required to validate these imaging findings in larger patient groups.

## AUTHORS’ CONTRIBUTIONS

All authors contributed to the study design, collection and analysis of the data, and preparation of the manuscript.

## Figures and Tables

**Table 1:** Patient demographics and symptoms.

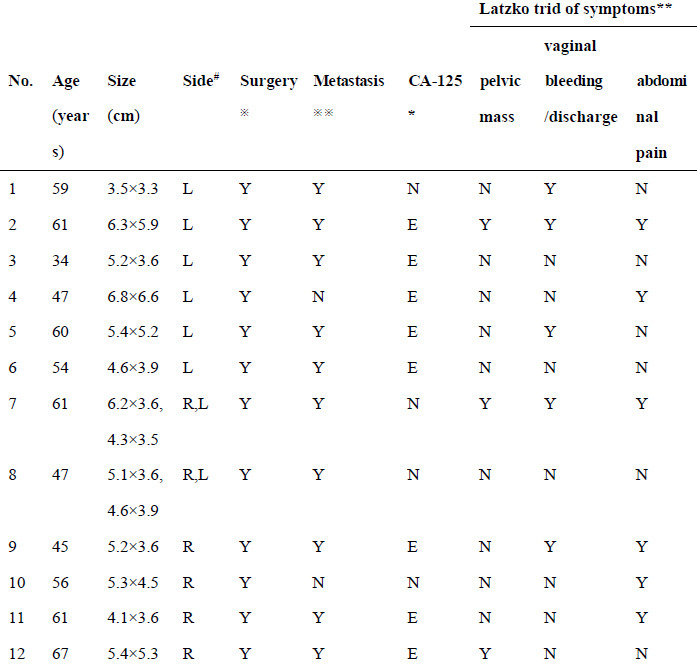

**Note: **# L=left; R=right; 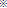 Y indicates that the mass was removed by operation; 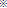

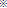
 Y indicates that the presence of lymph node metastasis; N indicates no metastasis; * N=normal range, E=elevated; ** Y=present, N=absent.

## Data Availability

The data and supportive information are available within the article.

## LIST OF ABBREVIATIONS

MPR: Multiplanar Reconstruction
MSCT: Multi-Slice CT
PFTC: Primary Fallopian Tube Carcinoma

## STANDARDS OF REPORTING

STROBE guidelines were followed.
